# Evaluation of a surgical service in the chronic phase of a refugee camp: an example from the Thai-Myanmar border

**DOI:** 10.1186/1752-1505-6-5

**Published:** 2012-08-06

**Authors:** Chathika K Weerasuriya, Saw Oo Tan, Lykourgos Christos Alexakis, Aung Kaung Set, Marcus J Rijken, Paul Martyn, François Nosten, Rose McGready

**Affiliations:** 1 Shoklo Malaria Research Unit (SMRU), PO Box 46 Mae Sot, Tak, 63110, Thailand; 2 Mahidol-Oxford Tropical Medicine Research Unit (MORU), Mahidol University, Bangkok, 10400, Thailand; 3 Centre for Clinical Vaccinology and Tropical Medicine, Churchill Hospital, Oxford, OX3 7LJ, UK

**Keywords:** Basic needs, Protracted, Chronic, Refugee, Surgery, Thai-Myanmar border

## Abstract

**Background:**

Published literature on surgical care in refugees tends to focus on the acute (‘emergent’) phase of crisis situations. Here we posit that there is a substantial burden of non-acute morbidity amenable to surgical intervention among refugees in the ‘chronic’ phase of crisis situations. We describe surgery for non-acute conditions undertaken at Mae La Refugee Camp, Thailand over a two year period.

**Methods:**

Surgery was performed by a general surgeon in a dedicated room of Mae La Refugee Camp over May 2005 to April 2007 with minimal instruments and staff. We obtained the equivalent costs for these procedures if they were done at the local Thai District General Hospital. We also acquired the list (and costs) of acute surgical referrals to the District General Hospital over September 2006 to December 2007.

**Results:**

855 operations were performed on 847 patients in Mae La Refugee Camp (60.1% sterilizations, 13.3% ‘general surgery’, 5.6% ‘gynaecological surgery’, 17.4% ‘mass excisions’, 3.5% ‘other’). These procedures were worth 2,207,500 THB (75,683.33 USD) at costs quoted by the District General Hospital. Total cost encountered for these operations (including staff costs, consumables, anaesthesia and capital costs such as construction) equaled 1,280,000 THB (42,666 USD). Pertaining to acute surgical referrals to District General hospital: we estimate that 356,411.96 THB (11,880.40 USD) worth of operations over 14 months were potentially preventable if these cases had been operated at an earlier, non-acute state in Mae La Refugee Camp.

**Conclusions:**

A considerable burden of non-acute surgical morbidity exists in ‘chronic’ refugee situations. An in-house general surgical service is found to be cost-effective in relieving some of this burden and should be considered by policy makers as a viable intervention.

## Background

**Figure 1:**
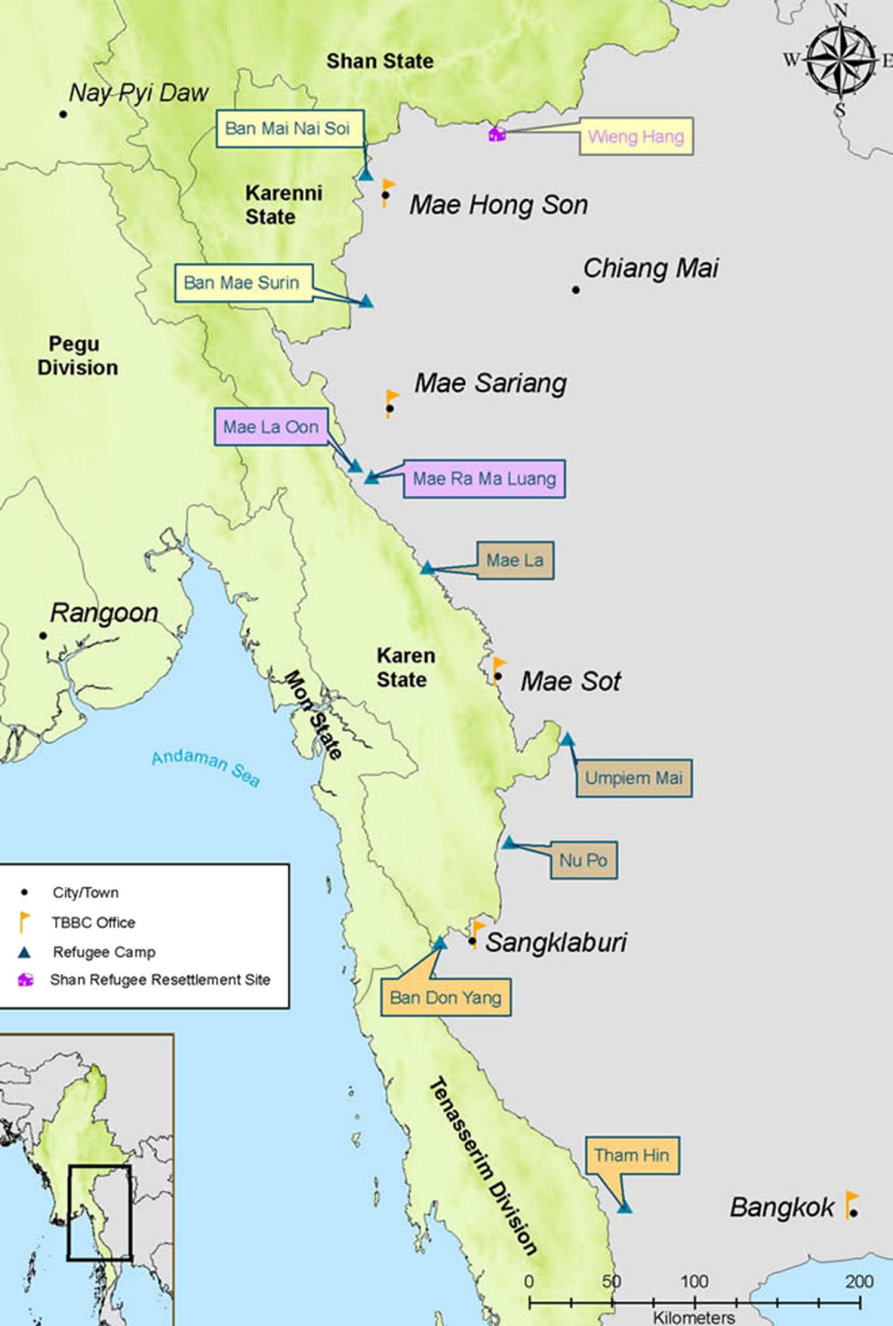
Refugee Camps along the Thailand-Myanmar Border (Image reproduced with permission from the Thailand Burma Border Consortium).

The published literature on surgery in refugee situations is concentrated on acute trauma in conflict situations [1-4] and reproductive health, the latter including female genital mutilation, refugee rights to abortion and family planning [5-9]. The focus is on the so called ‘emergent phase’ [10] of crisis situations which pertains to acute events (natural disaster, war, terrorist attack etc.). However, globally, a significant number of refugee populations reside in the protracted ‘chronic phase’ (either following an emergent event, or during prolonged low level conflict) – the surgical needs in these populations are poorly documented [10]. The Thailand-Myanmar border is one such protracted situation. Camps for displaced people from Myanmar (primarily of Karen ethnic origin) were established in 1984 (Figure 1). In Asia these refugee camps are second only to Afghanistan in terms of their chronicity. There are 9 camps ranging in size from 3,000 residents in Ban Mae Surin to 40,000 refugees in Mae La. Umpiem Mai and Mae Ra Ma Luang have approximately 16,000 residents each [11]. Distance and time to the nearest Thai Hospital varies between camps (30 mins to 8 hours) and according to the condition of roads, which deteriorate during the rainy season. Health care in Mae La is provided by Non-Governmental Organisations (NGOs) – initially by Médecins Sans Frontières (until 2005) and subsequently by Aide Médicale Internationale (AMI). The refugee situation in Mae La Camp is stable and the majority of morbidity is associated with infectious and chronic diseases; while war trauma and reproductive health problems exist, these are comparatively minor. One of the principle health issues is multi-drug resistant *Plasmodium falciparum* malaria – however significant improvements in this area have been made due to both early detection and control with mefloquine and artesunate combination therapy [12].

With respect to the management of surgical conditions, the policy in place at Mae La Camp has been to refer acute surgical conditions (e.g. acute appendicitis, strangulated hernia, bowel perforation) to secondary care (the local Thai District Hospital, which is Mae Sot General Hospital (MSGH)). Due to cost considerations, referrals are limited to emergencies or situations which require urgent surgical attention; emergency referrals in themselves incur a high cost as charges reflect out of hours staff mobilisation and theatre time. Camp residents are transported a distance of 60 km to the hospital.

Emergency surgery, particularly in the obstetric context, e.g. caesarean section, has been previously considered at Mae La, but thought not feasible for a number of reasons (apart from financial constraints). Firstly, there is a lack of trained staff to deal with complex cases, particularly anaesthetic care staff with knowledge of advanced airway control. Secondly, Mae La has no capacity for medium or long term storage of blood products and hence urgent blood for transfusion is not readily available. Donations are received from relatives and a known list of previous donors on a case by case basis. Pre-transfusion testing is limited to ABO grouping, an infection screen (malaria, Hepatitis B and HIV), haematocrit assessment and mixing of donor and recipient samples to visually assess for evidence of agglutination. Thirdly, in the specific context of obstetrics, many women who require caesarean sections (performed at MSGH) have newborns who subsequently require assisted ventilation which is not available in camp.

In contrast we posit that there is a considerable amount of unmanaged minor or non-acute morbidity which can potentially be relieved by surgical intervention. As such, we describe the minor (non-acute) surgical cases operated on over a 2 year period within the Mae La Refugee camp. These interventions were undertaken by a general surgeon (SOT) at the field site of the Shoklo Malaria Research Unit (SMRU) within Mae La Camp, in a small designated room for sterile procedures. The concurrent acute surgical referrals from Mae La to secondary care (MSGH) are also profiled.

In this article we attempt to quantify the unmet non-acute surgical needs in the “Chronic” Phase of a Refugee Situation and to estimate the value of providing surgery for non-acute needs early, and in-camp.

## Methods

The SMRU, founded in 1986, is a field based research unit with an aim to conduct research that directly impacts on the health of the local refugee and migrant population along the Thailand-Myanmar border. It also provides obstetric, paediatric and neonatal care to residents of the Mae La refugee camp. During 2005–2007 the Unit employed a general surgeon with special experience in obstetrics to assist locally trained midwives in the SMRU Delivery Unit located at Mae La Camp. Naturally the surgeon also attended to non-acute surgical conditions. As word spread in the camp more patients presented with non-acute surgical conditions seeking care. A minor surgery service was set up, wherein the general surgeon would (on a strictly part time basis) operate on camp residents deemed to have such needs. A minority of patients were referred by AMI, the main provider of health care in the camp. There was no advertising of the service.

### Pre-operative assessment

All patients had an initial consultation with the surgeon who assessed patients for suitability for both anaesthesia and surgery. Following this, if an operation was deemed suitable, patients were given a date for an elective operation. Cases were selected if suitable for day surgery – however, overnight stay facilities were available at the SMRU in-patient department if required. Consent was obtained verbally. Children were accompanied by and consented for by parents.

### Operating room, equipment and staff

One room at the SMRU field site was designated for operations. Equipment consisted of 10 dissecting forceps, 10 artery forceps, 6 pairs of scissors, 2 blade holders, 4 needle holders, 4 sponge forceps, 3 Babcock’s forceps, 3 Cusco vaginal specula, 2 Sims vaginal specula, 2 Vulsellum forceps, 2 hernia retractors and 2 Senn-Miller retractors. Instrument sterilization was via an industrial steam pressure sterilizer. Three midwives were trained as operating theatre assistants and four midwives were trained in anaesthetic care of the patient. Midwives received training in monitoring of vital signs, basic airway control (including bag and mask ventilation) and adult resuscitation, but not in intubation. All anaesthesia was ordered by and local anaesthesia delivered by the surgeon. Midwives provided general anaesthesia (ketamine) but only under supervision of the surgeon.

### Anaesthesia

Local anaesthesia was performed with 2% lignocaine for local infiltration and/or regional block, general anesthesia with 2–5 mg/kg IV ketamine sulphate and spinal anesthesia with 5% heavy bupivicane followed by post-operative rest for 8 hours in the supine position. Patients undergoing general anaesthesia were maintained nil by mouth the night prior to the operation. There was no pre-anesthetic medication and all patients requiring post operative analgesia were treated as required. There was no routine use of peri-operative antibiotics. Their use was restricted to operations complicated by prolonged handling of tissue, prolonged operative time, wound dehiscence and sepsis. Dressings were changed routinely on the 3^rd^ post-operative day and in the majority of cases sutures were removed at day 7. All patients were reviewed at day 7 and later again if required. Patients were asked to come back to the surgeon if they experienced problems post-surgery. Specimens for histopathological analysis, kept to a minimum due to budget constraints, were sent to MSGH.

### Acute surgical referrals to district hospital

In order to compare the profile of general surgical operations performed by the surgeon at Mae La Camp to the profile of operations performed by Mae Sot General Hospital (MSGH) on Mae La Camp patients, we compiled a list of “Acute Surgical Referrals” to MSGH. Data were supplied by AMI via the Mae La Camp “referral logbook”; however complete data were only available for the period of September 2006 to December 2007 (14 months). We also acquired the costs for each of these referrals to perform cost-analysis.

In this context, an “Acute Surgical Referral” is defined as a referral to MSGH which satisfies the following conditions: 1) it results in the first operation for indications where surgery is acutely necessary to resolve or relieve the indication and 2) the operative need could not be met in the refugee camp.

The definition includes the first operative interventions for 1) acute indications (e.g. acute abdomens), 2) acute-on-chronic indications, for example operative intervention for chronic osteomyelitis, chronic inguinal hernia and uterine myoma removal and 3) obstetric operations (but these are excluded from this analysis). It excludes follow up operative interventions, for example post-fracture plate removal and second (follow-up) dilatation & curettage operations for choriocarcinoma. Finally, we have grouped acute surgical referrals into “preventable” and “non-preventable” on the basis that “preventable” referrals were those for which intervention might have been performed earlier as non-acute cases by the in-house surgical service.

### Analysis

In order to estimate the value of the in-house surgical service, we compared the cost of procedures done at the camp to the expected cost had they been undertaken at MSGH. To perform this comparison we obtained a comprehensive list of prices from Mae Sot General Hospital for each type of procedure performed at Mae La camp. These prices were specifically those charged by the hospital for non-acute uncomplicated cases, as were being operated on at Mae La. We also calculated the cost-savings to be anticipated by preventing acute referrals to MSGH, by operating for these indications in-house at an earlier, non-acute stage. Conversion of Thai Baht (THB) to US Dollars (USD) is performed at a rate of 30 THB: 1 USD in this analysis. Non-normal data are described using median [range].

## Results

### Non-acute surgical burden

A total 855 operations were performed on 847 patients between May, 2005 and April, 2007 at SMRU; 8.6% (73/847) of these patients were sent by AMI and the remainder self presented to SMRU. There was a variable case mix (Table 1) dominated by sterilisation (60.1% of cases; n=514) of both female (477) and male (n=37) patients. The remaining cases were grouped into “general surgery” (13.3%, n=114), “gynaecological surgery” (5.6%, n=48), “mass excisions” (17.4%, n=149) and “other” 3.5% (n=30). The other subgroup of gynaecological surgery was predominantly tubal insufflations with dilatation and curettage (indicated for primary infertility), cervical polypectomy and a variety of other minor operations; of note, there was a single cervical amputation procedure.

**Table 1:** Elective surgery by operative group in Mae La refugee camp, May 2005-April 2007

**Surgical Procedure**	**No. of cases**	**Percentage of total**	**Cost per procedure (THB)**	**Totals (THB)**	**Totals (USD)**
*Sterilisation*					
Tubal ligation	477	55.8	2,500.00	1,192,500.00	39,750.00
Vasectomy	37	4.3	2,000.00	74,000.00	2,466.67
*General Surgery*					
Hernia repair	51	6.0	5,000.00	255,000.00	8,500.00
Hydrocele repair	21	2.5	3,000.00	63,000.00	2,100.00
Male Circumcision	21	2.5	2,000.00	42,000.00	1,400.00
Haemorrhoidectomy	21	2.5	3,500.00	73,500.00	2,450.00
*Gynaecological surgery*					
Surgical Perineal repair	6	0.7	†	23,000.00	766.67
Anterior colporrhaphy and posterior colporrhaphy	2	0.2	2,500.00	5,000.00	166.67
Posterior colporrhaphy alone	13	1.6	4,000.00	52,000.00	1,733.33
Salpingo-oophorectomy	2	0.2	6,000.00	12,000.00	400.00
Other (gynaecological)	25	2.9	†	100,000.00	3,333.33
*Mass excisions*					
Lipoma	38	4.4	2,500.00	95,000.00	3,166.67
Cystic lesion	47	5.5	2,500.00	117,500.00	3,916.67
Corn and/or papilloma	9	1.1	2,500.00	22,500.00	750.00
Tumour biopsy	8	0.9	2,500.00	20,000.00	666.67
Breast lump excision	12	1.4	2,500.00	30,000.00	1,000.00
Neck gland biopsy (including thyroid)	12	1.4	‡		
Granuloma	4	0.5	2,500.00	10,000.00	333.33
Nasal polyp	3	0.4	4,500.00	13,500.00	450.00
Plastic surgery	16	1.9	†	26,500.00	883.33
*Other*	30	3.5		43,500.00	1,450.00
**Totals**	**855**	**100**		**2,270,500.00**	**75,683.33**

† Totals for these operation groups are calculated by summing the cost of the individual procedures. Cost data for some of the individual procedure types in these groups were not available. ‡ Cost data for these operations were not available.

**Figure 2:**
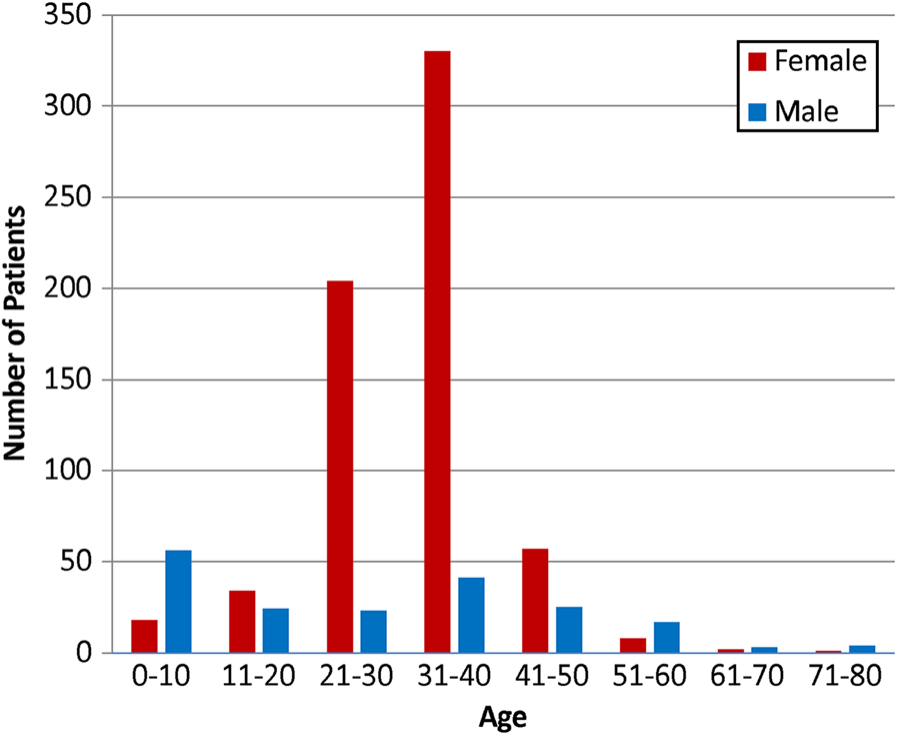
Age/sex distribution of patients operated on in Mae La Refugee camp, May 2005 – April 2007.

The age distribution of the patients was skewed (Figure 2) with a predominance of male patients at the extremes of age and of females in the reproductive age group. The median [range] age and parity of women who had sterilization by mini-laparotomy was 33 [23–50] years and 5 [2-13] children. 41.2% (352/855) of procedures required general anaesthesia, 8 spinal anesthesia and the remaining operations were performed under local anesthesia.

Hernias included both congenital inguinal hernias and hernias in adults causing discomfort. The majority of surgery in the “mass excisions” category was indicated for benign lesions as would be encountered on any minor surgery list. Due to prolonged neglect a number of these were of impressive proportions, e.g. an 18 x 12 cm lipoma, which was impeding normal daily function and impairing quality of life. “Mass excisions” have been detailed in Table 1. The category of “other” included a mix of operations including incision and drainage of abscesses (both deep and superficial), removal of foreign bodies and minor colorectal surgery (anal polypectomy and fistulectomy).

Twenty two biopsies were sent for histopathological analysis which confirmed the macroscopic diagnosis of cancer in six cases; three were tuberculosis and a further two that did not look cancerous that were in fact malignant. Circumcision (n=21) was carried out following confirmed, usually recurrent, urinary tract infection. Haemorrhoidectomy was performed in patients reporting recurrent rectal bleeding, including 9.5% (2/21) who had required transfusion for the bleeding. Over the entire two year period, there was a median [range] of 8 [0 – 18] operations per week.

### Post-operative complications

Thirty-two operations (3.7%) were followed by complications including 17 cases of wound infection (including abscess), 1 case of wound dehiscence and 12 cases of granuloma formation. These complications were managed in-house at Mae La and did not require further referral to Mae Sot District General Hospital. One supramyeloid dermoid cyst and 1 ganglion case recurred and were re-operated with no further recurrence. No deaths occurred as a complication of surgery.

### Acute surgical referrals to Mae Sot hospital

There were a total of 304 Acute Surgical Referrals made to Mae Sot General Hospital from Mae La Refugee Camp during the 14 month period between September 2006 and December 2007 (Table 2). Most, 75% (227/304) of these were non-obstetric, and the majority, 91% (207/227) of these referrals were for “General” surgical indications; “General” here encompasses abdominal surgery, wounds/trauma (including fractures), surgery for infection (chronic osteomyelitis, joint infection and soft tissue infection), mass excision (both malignant and non-malignant; these indications overlapping with “mass excisions” surgery in-house at Mae La Refugee Camp) and a small number of miscellaneous operations (1 vascular operation – to relieve acute arterial occlusion of the leg, and 3 urological interventions – to relieve urethral stones). Surgical intervention for fractures and soft tissue infection (including abscess) were the two largest single indications for “General” surgery (80 and 29 referrals, respectively).

**Table 2:** Acute Surgical Referrals (September 2006-December 2007) by Referral Speciality

**Referral Speciality**	**No. of referrals**	**Percent of total**
General	207	68.1
Gynaecology	15	4.9
Obstetrics	77	25.3
Opthalmology	5	1.6
Total	304	100.0

In addition to “General” surgery, there were also 15 referrals for Gynaecological surgical intervention, and 5 referrals for Ophthalmological Surgery**.** The gynaecological indications included ovarian masses (n = 6), uterine myoma (n = 3), procidentia (n = 2) and single cases of cystorectocoele, uterine malignancy, rectovaginal fistula and adenomyosis. The ophthalmological procedures included repair of two ruptures corneas, an anterior lens dislocation, a nasolacrimal duct obstruction and a cataract operation.

The total cost of the 227 non-obstetric Acute Surgical Referrals to MSGH over the 14 month period was 3,885,219.00 THB (129,507 USD), with a median [range] cost per referral of 10,702.00 [144.00 – 338,791.00] THB or 356.73 [4.80 – 11,293.00] USD.

### Preventable acute surgical referrals

The range of indications referred to MSGH was larger than that handled in-house at Mae La. A number of cases referred to MSGH could potentially have been handled in the Mae La service at a non-acute stage: hernia repair (n = 9), hydrocoele repair (n = 1), anterior-posterior repair (for cystorectocoele; n = 1), salpingo-oophorectomy (for ovarian masses; n = 6), nasal polyp excision (n = 2) and abscess excision (in the “Other” category of in-house operations and “General” category of acute surgical referrals to MSGH; n = 12). These referrals were considered “preventable”, had intervention taken place earlier and are detailed in Table 3; they incurred a total cost of 356,411.96 THB (11,880.40 USD) at MSGH.

**Table 3:** (Potentially) preventable referrals to Mae Sot General Hospital (02 September 2006 – 31 December 2007 )

**Operation**	**No. of operations**	**Mean (SD) cost of procedure at MSGH (THB)**	**Total costs of procedures at MSGH (THB)**
General			
Hernia repair	9	11,284.00 (5,582.51)	101,556.00
Hydrocoele repair	1	7,225.00	7,225.00
Mass Excisions			
Nasal polyp	2	11,414.50 (603.16)	22,829.00
Gynaecology			
Cystorectocoele (Anterior-Posterior repair)	1	15,086.00	15,086.00
Salpingo-oophorectomy	6	14,596.50 (3,451.52)	87,579.00
Other			
Abscess excision	12	10,178.08 (3,844.95)	122,136.96
**Total**	**31**		**356,411.96**
**Total cost in USD**			**11,880.40**

### Value of in-house surgery

We estimated the value of surgery performed by the Mae La camp in-house surgical service by obtaining typical pricing information for each type of procedure from Mae Sot General Hospital. Over two years, this value was equivalent to 2,207,500.00 THB (75,683.33 USD). The procedures which contribute most to this total, by virtue of their frequency, are sterilisations and hernia repairs. In terms of the costs of running the surgical service: staff salaries (540,000 THB (18,000 USD)) and consumables (in-patient stay, anaesthetic etc.) and construction and maintenance (100,000 THB (3,333 USD)) amount to a cost of 640,000 (21,333 USD) per annum. The total cost incurred by the Mae La camp in-house surgical services was therefore 1,280,000 THB (42,666 USD) over two years.

## Discussion

We have profiled the non-acute surgical procedures performed in Mae La Refugee camp over May 2005 – April 2007. Excepting the dominance of sterilizations (approximately 60% of all procedures) the remaining operations are of a similar nature to that expected on a minor or general surgery list in an equally sized non-refugee community. The dominance of sterilizations is not unexpected given the unmet need in this area.

The profile highlights the need for routine minor operations: apart from sterilizations, mass excisions (particularly lipoma and cystic lesions) and hernia repair were in high demand. Within the gynaecological operations, intervention for uterine prolapse was prominent. Surgery for infection (abscesses in the “Other” category) was also common. Procedures for these indications are not necessarily complex, and as demonstrated here, can be carried out with minor complications and good outcome. While we could not find a report on complication rates for this type of mixed general surgery, it is anticipated that such a rate would be low, and similar to that which we have found given that this type of elective and pre-emptive surgery is generally not associated with many complications. However, under-reporting of complications may be due to recording errors, misattribution (e.g. not attributed to post-operative state) or by patients presenting to the other health facility in the camp (run by AMI). Finally, there were no recorded cases of complications related to anaesthesia.

The number of procedures conducted over two years was large, probably due to backlog and a policy of referring only acute cases. Aside from direct symptom relief and quality of life gains (e.g. from excision of large, debilitating masses) many of the procedures performed have additional long term benefits. Sterilization (with reduced subsequent risks of grand multigravidae complications such as post partum haemorrhage), hernia repair (reduced strangulation risk) and haemorroidectomy (reduced risks associated with blood transfusion) are a few examples of this.

In the context of healthcare in resource poor settings, surgery has been presumed to be an expensive intervention and largely sidelined for more prevalent and easily managed causes of mortality and morbidity (e.g. infectious diseases, reproductive health needs) [13]. Nonetheless, there is now increasing recognition that surgical conditions account for a significant proportion of global morbidity, estimated at 11%, and that this burden is disproportional to the developing world [14]. Evidence is emerging that basic surgical care is cost-effective in terms of averting the loss of Disability Adjusted Life Years (DALYs) [15,16]. However it must be borne in mind that this new evidence is from small hospitals catering to native populations, not from chronic refugee situations, and is generally biased in favour of acute, potentially life threatening surgical indications. Similarly, while evidence is mounting that the surgical needs of civilians in conflict situations are not primarily war-injury related, and that a substantial proportion is attributable to accidental injury and infection [17], this evidence is still confined to the “Emergent” phase of crisis. To our knowledge, our study is the first to specifically address non-acute surgery in the “Chronic” phase.

In the specific case of a chronic refugee situation, there are three, broad, possible solutions to managing non-acute surgical morbidity: 1) referral of cases to specialist surgical services (i.e. a District Hospital), 2) the use of visiting specialist surgeons to perform series of operations *en bloc* for specific indications, or 3) the use of a general surgeon, or some combination of these. It is cost-prohibitive to refer all non-acute surgical cases to secondary care. This leads to the current policy of referring only acute or urgent cases, while non-acute conditions remain largely unmanaged. The foreseeable problems with this approach are a large pool of unmanaged morbidity (as demonstrated) and the potential for conversion to acute complicated situations. However there is little data to support or refute the hypothesis that preventive surgery in this context (e.g. for uncomplicated hernia) will be cost-effective in reducing expensive, acute complications [14].

Conversely, the use of visiting specialist surgeons has been demonstrated to be a highly effective strategy in some situations; the most notable local example of this is the provision of cataract surgery along the Thai-Myanmar border. A visiting ophthalmologist performs cataract surgery during two-week blocks, two to three times a year with up to 600 procedures per annum. This program has been highly successful in reducing or reversing visual impairment in the refugee population. However, this success relies on several factors: long-term commitment and regular visits made by the team of volunteers, narrow indications for which the surgeons operate, a high level of local staff training to identify and pre-operatively counsel and prepare patients (prior to the arrival of the surgeons), good cooperation between the eye team and local health bodies, and no potential for acute conversion to a complicated condition. Additionally, prevalence of cataract is relatively high, contributing to the cost-efficiency of the program. It is difficult to apply this strategy for the range of conditions we have identified in our study – no indication other than cataract is of sufficient prevalence to justify a visiting specialist for that indication alone. Furthermore, there is a lack of trained staff to adequately identify and select patients outside the surgical window. Finally, some of the indications found here have the potential for conversion to acute situations with prolonged wait.

In the limited cost analysis that was possible for this data, the use of a general surgeon to perform a limited range of non-acute operations was most favourable. The in-house surgical service of Mae La Camp performed general surgery worth approximately 2,200,000 THB (75,000 USD) over a two year period, calculated using cost data for those procedures obtained from MSGH. Concurrent costs of the service were approximately 1,300,000 THB (43,000 USD), leading to a cost saving of approximately 900,000 THB (~32,000 USD) compared to if these cases were referred onwards. The estimated cost of the Acute Surgical Referrals to MSGH over 14 months (3,800,000 THB [130,000 USD]) is substantial. Approximately 350,000 THB (11,800 USD) of this total was attributable to ‘preventable’ referrals.

The calculations above are conservative because we were unable to obtain hospital cost data for some of the procedures performed in-camp; the value of these procedures has been excluded. Further, the beginning of the period over which ‘preventable’ referral costs are estimated is subsequent to the beginning of the surgical service by more than a year and it is likely that the acute complication (and therefore referral) rate was somewhat lower than prior to the institution of the service. Finally, running costs, particularly staffing cost, of the service are an overestimate. In the first instance, the surgeon and midwives were employed to perform an obstetric service; the general surgical service took a secondary role and therefore total staff costs cannot be attributed solely to the latter.

As discussed, a backlog of cases likely contributes to the high volume encountered here. It is possible that over time, the rate of new referrals would have diminished to, or reached a plateau at, a level where the in-house surgical service was no longer cost-effective but this trend was not observed in the time period. Conversely, as the service was not advertised – it relied mostly on word of mouth within the camp – it is probable that only a proportion of people with potential surgical problems presented; other patients would have never heard about the service. As the surgical service was terminated in 2007 (the surgeon left for another position), we have no further data with which to analyse these hypotheses. An option to extend the efficiency of a surgical service such as this might include pooling patient populations from multiple refugee camps, e.g. transporting patients to Mae La refugee camp from neighbouring camps in the same region. However this solution must be considered within the local context – logistical and security factors increase the complexity of transporting refugees (and staff, e.g. midwives) between sites in host nations, particularly along the Thai-Myanmar border.

In surgical care provision, adjunctive considerations include anaesthetic and operative complications and availability of adequate post-operative care. As noted above, we found an acceptably low rate of operative and post-operative complications, with no anaesthetic complications. Contributing factors to this a caseload of consisting of relatively minor procedures and a low threshold for exclusion of cases during pre-operative assessment. The latter was due to a lack of material and human resources to deal with complex cases, as well as relatively basic anaesthetic and resuscitation facilities. In terms of anaesthetic services and risk, midwives providing care were trained only in basic airway management. While this level of anaesthetic care is a cost effective solution adequate for simple, low risk procedures, it was considered inadequate for cases with a high risk of needing resuscitation. Anaesthetic care is therefore likely to be a limiting factor in any such surgical service. Furthermore, lack of blood products limits the scope of feasible procedures.

A final consideration is the justification for elective surgical service provision in chronic refugee situations, given that such services are often lacking at District or Regional level in many low income countries. Refugee situations experience factors unique to them – chief amongst these is reduced freedom of movement. Logistical and security factors impede movement (and transport) of refugees within host nations – these factors influence referral decision making. The population is often reliant entirely on the profile of services provided by NGOs and has no freedom of choice *per se*. While a comparatively sized population in a low income country will likely suffer from under capacity for elective surgery, these populations have (albeit often limited) recourse via referral either laterally to other providers or upward to Regional centres.

When considered together: the burden of non-acute surgical morbidity, the cost to NGOs of referral (both non-acute cases and acute conversions) and the relatively low cost of operating a simple minor surgical service within the camp, the argument for such a service appears positive. Further research is required into feasibility of the delivery of such surgical care: with increasing specialisation in surgery, the availability of general surgeons with requisite skills and experience to manage this range of conditions may be in question. Moreover, while some of the conditions managed in-house in our context are presumed to be beyond the capacity of a non-surgeon physician, surgical task shifting has been deployed safely and cost-effectively elsewhere [18,19]; further study is required as to what extent such strategies can be employed in chronic refugee situations.

## Conclusions

A considerable burden of non-acute surgical morbidity exists in ‘chronic’ refugee situations. A simple, in house general surgical service is likely to be cost effective in relieving some of this morbidity and improving quality of life. Policy and decision makers should consider expanding the role of surgery in refugee situations beyond acute surgical emergencies,

## Abbreviations

AMI: Aide Médicale Internationale; DALY: Disability Adjusted Life Year; MSGH: Mae Sot General Hospital; SMRU: Shoklo Malaria Research Unit; TBBC: Thailand Burma Border Consortium; THB: Thailand Baht; USD: United States Dollar.

## Competing interests

The authors declare that they have no competing interests.

## Authors’ contributions

SOT, PM and RM developed the idea for the manuscript. SOT and PM performed the surgical work. SOT, LCA, AKS and RM organized the data collection. CKW, MJR and RM analyzed the data. CKW, MJR, FN and RM drafted the manuscript with all authors contributing to the final version. All authors read and approved the final manuscript.
