# Erratum to: Evaluation of a surgical service in the chronic phase of a refugee camp: an example from the Thai-Myanmar border

**DOI:** 10.1186/s13031-016-0092-7

**Published:** 2016-08-01

**Authors:** Chathika K. Weerasuriya, Saw Oo Tan, Lykourgos Christos Alexakis, Aung Kaung Set, Marcus J. Rijken, Paul Martyn, François Nosten, Rose McGready

**Affiliations:** 1 Shoklo Malaria Research Unit (SMRU), PO Box 46, Mae Sot, Tak 63110 Thailand; 2 Mahidol-Oxford Tropical Medicine Research Unit (MORU), Mahidol University, Bangkok, 10400 Thailand; 3 Centre for Clinical Vaccinology and Tropical Medicine, Churchill Hospital, Oxford, OX3 7LJ UK; 4 Aide Médicale Internationale (AMI), Mae Sot, Tak Thailand

## Erratum

Unfortunately, the original version of this article [1] contained an error.

Lykourgos Christos Alexakis was incorrectly assigned affiliation “Mahidol-Oxford Tropical Medicine Research Unit (MORU), Mahidol University, Bangkok 10400, Thailand” (affiliation number 2), instead of “Aide Médicale Internationale (AMI), Mae Sot, Tak, Thailand” (affiliation number 4 in this erratum).

All affiliations are correctly included in full in this erratum.
